# 3D Object Detection under Urban Road Traffic Scenarios Based on Dual-Layer Voxel Features Fusion Augmentation

**DOI:** 10.3390/s24113267

**Published:** 2024-05-21

**Authors:** Haobin Jiang, Junhao Ren, Aoxue Li

**Affiliations:** 1 Automotive Engineering Research Institute, Jiangsu University, Zhenjiang 212013, China; 2 School of Automobile and Traffic Engineering, Jiangsu University, Zhenjiang 212013, China; renjh79@163.com (J.R.); liax@ujs.edu.cn (A.L.)

**Keywords:** object detection, voxel feature fusion, channel attention mechanism, multimodal fusion

## Abstract

To enhance the accuracy of detecting objects in front of intelligent vehicles in urban road scenarios, this paper proposes a dual-layer voxel feature fusion augmentation network (DL-VFFA). It aims to address the issue of objects misrecognition caused by local occlusion or limited field of view for targets. The network employs a point cloud voxelization architecture, utilizing the Mahalanobis distance to associate similar point clouds within neighborhood voxel units. It integrates local and global information through weight sharing to extract boundary point information within each voxel unit. The relative position encoding of voxel features is computed using an improved attention Gaussian deviation matrix in point cloud space to focus on the relative positions of different voxel sequences within channels. During the fusion of point cloud and image features, learnable weight parameters are designed to decouple fine-grained regions, enabling two-layer feature fusion from voxel to voxel and from point cloud to image. Extensive experiments on the KITTI dataset demonstrate the significant performance of DL-VFFA. Compared to the baseline network Second, DL-VFFA performs better in medium- and high-difficulty scenarios. Furthermore, compared to the voxel fusion module in MVX-Net, the voxel feature fusion results in this paper are more accurate, effectively capturing fine-grained object features post-voxelization. Through ablative experiments, we conducted in-depth analyses of the three voxel fusion modules in DL-VFFA to enhance the performance of the baseline detector and achieved superior results.

## 1. Introduction

Intelligent networked vehicles represent an essential development direction in today’s cutting-edge science and technology, significantly impacting smart city road transportation, modern logistics, and other areas [1,2,3]. The environment perception system serves as the foundation and guarantee for the safe operation of intelligent vehicles on the road. Real-time and accurate object detection stand as one of the crucial functions of intelligent vehicles, allowing them to perceive the surrounding environment. The precise judgment of distance, position, attitude, and other information regarding surrounding objects remains a classic problem in 3D object detection for autonomous driving [4]. Within the intelligent vehicle environment sensing system, cameras provide rich semantic information such as color and texture, while LiDAR offers accurate depth information. Effectively fusing these two types of heterogeneous data constitutes a current research hotspot and challenge. Due to the development of artificial intelligence technology, the importance of neural networks in object detection is self-evident. As a powerful machine learning tool, neural networks, through deep learning techniques, can extract rich features from complex image or video data and use these features to identify and locate objects in images. The quality of its design and training directly affects the performance and accuracy of object detection systems. Therefore, in research and practice, a thorough understanding of the structure, parameter settings, and training strategies of neural networks is crucial for improving the effectiveness of object detection systems.

With the rapid development of deep learning technology, many classical multimodal fusion frameworks have emerged in recent years [5,6,7,8]. Multi-view 3D networks (MV3D) [9] takes in the front view of the laser point cloud, the bird’s-eye view of the laser point cloud, and an RGB image for feature extraction. It employs regression on the bird’s-eye view features to derive the initial 3D boundaries of objects. These boundaries are then projected onto various planes to extract regional features. A feature fusion network is utilized to integrate the information from the three input features. Finally, multitask prediction is employed to obtain relevant object information. Based on MV3D, the Aggregate View Object Detection networks (AVOD) [10] only utilize the network of laser point cloud aerial views and RGB images, generate region proposals using the Region Proposal Networks (RPN) [11], and enhance the detection of small objects by up-sampling feature mapping using a feature pyramid extension network. However, this approach only partially utilizes the depth information of the laser point clouds. PointPainting [12] sequentially fuses the image semantics of the objects with the input point cloud to achieve an increase in point cloud dimensionality and feeds this richer point cloud information into a pure point cloud-based objects detection network, enabling the performance of the original network to be improved. The multimodal virtual point network (MVP) [13] uses an image segmentation mask to generate virtual points to complement the sparse point cloud and feeds the dense point cloud into CenterPoint [14] to complete the detection. However, these research methods rely too much on reliable 2D detection results, and it is challenging to detect objects that are not seen in the image, even if they have apparent features in the point cloud. MVX-Net [15] projects voxel features from the point cloud into the image feature map and uses the Region Proposal Network (RPN) to perform 3D detection on projected and voxel features. Although this method can reduce information loss due to view changes, it loses many local details during voxelization to extract features and cuts the connection between objects. Thus, it can be inferred that by optimizing the fusion of point cloud and image features, the detection network’s capability to extract crucial local information of the objects is enhanced.

**Figure 1:**
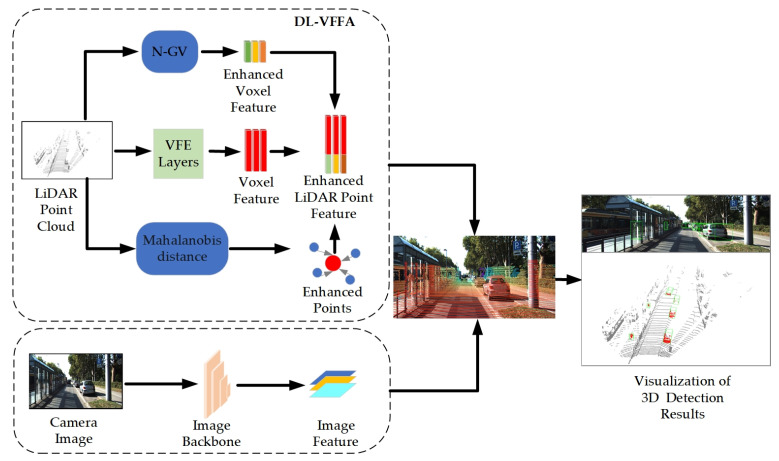
Diagrammatic representation of the general architecture of DL-VFFA. The DL-VFFA architecture consists of three main stages: (1) extraction of camera features using the image feature extraction network ResNet [19]; (2) acquisition of enhanced voxel features using an improved voxelized feature extraction network; (3) a dual semantic feature association module (cascading 3D point cloud location information with semantic information of the image).

Inspired by the above method, in order to preserve more local details and connections between objects during the voxelization process, we propose a dual-layer voxel feature fusion augmentation network to capture the finer feature of voxels. By associating the point cloud information with the underlying feature map of the image in the early fusion stage, constructing local voxel and global voxel units, extending the pooling layer in the two-dimensional convolution to increase the dimensionality of the head layer, and dynamically calculating the standard deviation of each position so that it can adaptively adjust the Gaussian deviation matrix according to the characteristics of the input data as a way of correcting the relative positional encoding of the corresponding voxel features in the attention matrix, the variant structure of spatial attention is used to capture the correlation of different channels better. A sparse 3D convolutional network [16] generates a weight for each portion of voxels in the point cloud space to efficiently select voxelized features, which improves network detection performance by fusing voxel-level semantic information [17]. The channel attention mechanism is improved when aggregating image and point cloud information and the generated attention weights are truncated to ensure that the model does not rely too much on certain local information so that the detection network adjusts the weight coefficients between point cloud and image features when facing different detection scenarios, and realizes the adaptive splicing of the two elements [18]. The joint feature mapping is obtained by projecting the body mass centroids into the image features through these two modules. This ensures an accurate correlation of image and point cloud features and reduces the dependence on high-resolution LiDAR point clouds. The network architecture of DL-VFFA is shown in Figure 1.

This paper proposes a novel fusion framework, and the main contributions are summarized as follows:

(1) During point cloud voxelization, fixed grid settings may lead to loss of local fine-grained features. We employ the Mahalanobis distance to link boundary point information for each voxel, yielding voxel feature mappings that are better aligned with local object information.
(2) We constructed neighborhood voxel and global voxel modules (N-GV) based on the voxelization network. We improved the attention Gaussian deviation matrix (GDM) to compute relative position encodings corresponding to voxel features.
(3) During the fusion stage of point cloud and image features, we designed a new set of learnable weight parameters (LWP), thereby expanding and enhancing the feature information of key points in the attention fusion module.

## 2. Related Works

### 2.1. LiDAR-Based 3D Object Detection

Three-dimensional object detection methods based on LiDAR point clouds can be broadly classified into three categories: the first category is based on the original point cloud processing, which is to extract features directly from the actual point clouds. Qi proposed PointNet [20] with cross-generation significance, where the input data is the original disordered point cloud, and designed an end-to-end deep learning network for point clouds. Subsequently, PointNet++ [21] was proposed, which extracts local attribute features by feeding each point set into a local point network. It further enhances recognition accuracy by progressively encoding higher-level features through layer-by-layer hierarchal processing. The second category is based on point cloud projection, which projects an unstructured point cloud in 3D space onto a plane, and through data processing, its dimensionality is reduced to two dimensions, and then processed using two-dimensional convolution. The third category is the spatial voxel method, which draws inspiration from the concept of two-dimensional pixels in image processing. It normalizes the point cloud space by rasterizing it, encodes the features of the laser point cloud within the grid, then utilizes a 3D sparse convolutional network to sequentially extract the sampled voxel features. Ultimately, it outputs the category of the objects. Voxel-Net [22] voxelizes the point cloud space using Voxel Feature Encoding (VFE) to learn the feature representation of each 3D voxel, thus implementing an end-to-end deep neural network tailored for point clouds. Subsequently, some scholars improved Voxel-Net and proposed networks such as Second [23] and PointPillars [24], which not only preserved the shape of objects to the maximum extent but also solved the problem of low efficiency of direct convolution due to the sparsity of point clouds. At present, the spatial voxel method has the problem of wasting computational resources due to the sparsity of laser point clouds that leads to many empty voxels, and there are also problems such as information loss in the feature-encoding process.

### 2.2. Multi-Modal-Based 3D Object Detection

LiDAR provides accurate depth information for advanced vehicles. However, LiDAR cannot capture the color and texture of objects in the scene, whereas cameras can provide rich semantic information but struggle with depth estimation. These complementary features make LiDAR and camera fusion the dominant sensor selection scheme in detection today [25,26,27]. The existing multimodal fusion models fall into three main categories: data layer fusion [28,29,30], feature layer fusion, and decision-making layer fusion [31]. These categories refer to different approaches for combining sensor data. In recent years, scholars have explored these three modes and attempted to combine them, known as deep fusion or heterogeneous fusion. Deep fusion [32,33,34,35] combines feature-level data from LiDAR with data-level or feature-level data from images. PointFusion [36] networks utilize convolutional neural networks (CNN) and PointNet models to process both image and LiDAR data, generating 3D proposals based on the extracted features. This study expands on various LiDAR 3D detection networks and demonstrates that augmenting LiDAR data with classification information can improve detection scores. The FusionPainting [37] network acquires semantic information of 2D images and 3D LiDAR point clouds based on 2D and 3D segmentation methods, then uses an attention mechanism to fuse the two semantics. The fused semantic labels drawn by the point cloud marked with fused semantic labels are finally sent to the 3D detector to obtain 3D results.

## 3. Methods

DL-VFFA contains two important modules: the voxel features fusion module and the LiDAR and image feature fusion module based on the improved channel attention mechanism.

### 3.1. Voxel Feature Fusion Module

**Figure 2:**
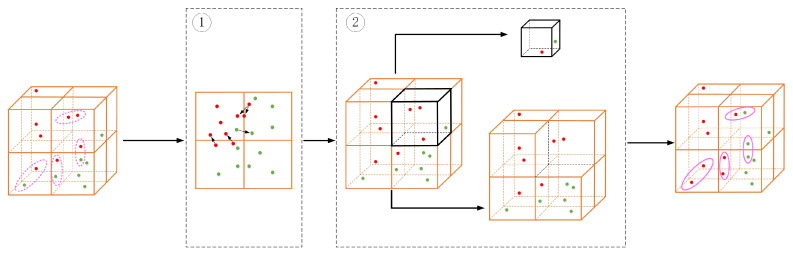
The (**Subfigure1**) represents the use of Mahalanobis distance to associate boundary point clouds between different voxel units, while the (**Subfigure2**) represents the constructed neighborhood voxels and global voxels. The pink dashed ellipse and pink solid ellipse respectively represent the point clouds before and after association. The red and green point cloud clusters are two objects, respectively, and the point clouds are segmented into other voxels during voxelization to extract features. The Mahalanobis distance determines the class of the point cloud by calculating the Mahalanobis distance between the two points clouds, which complements the features of each voxel.

To fully exploit the local information of each voxel grid, the similarity between the boundary points in the current voxel grid and the points in its neighborhood voxel grid is determined by the Mahalanobis distance [38] to reduce the loss of local information at the early fusion stage. The specific method is shown in Figure 2.

The Mahalanobis distance is a method proposed by Indian statisticians to calculate the covariance distance between two points. To define the Mahalanobis distance between two boundary points, the specific calculation formula is shown in Equation (1):

(1) $$D_{P}=\left(P_{m},P_{n}\right)=\sqrt{{\left(P_{m}-P_{n}\right)}^{T}S^{-1}\left(P_{m}-P_{n}\right)}$$

$D_{P}$ is the Mahalanobis distance between two boundary points, and $S^{-1}$ is the covariance matrix between two boundary points.

**Figure 3:**
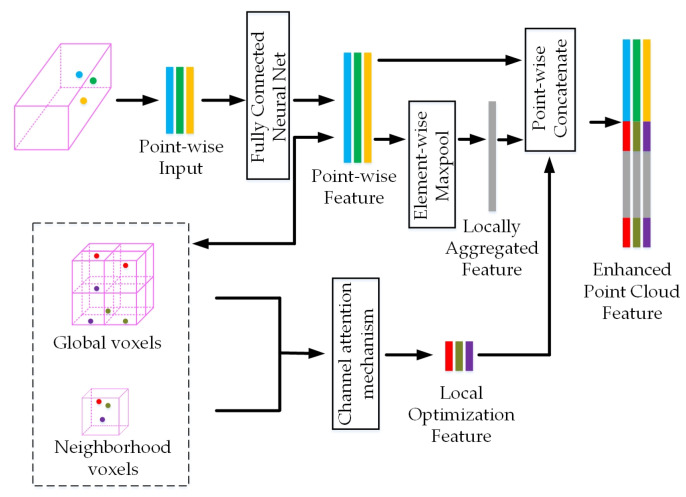
Voxel feature fusion module. When the original voxelized network extracts features point by point, NV and GV modules are added, and optimized voxel features are obtained after aggregating both features by the improved channel attention mechanism and are finally spliced with the original voxelized features.

In order to fully utilize the fusion of features from all voxels, we use the improved Voxel-Net as our voxel fusion framework. Additionally, we integrate the neighborhood voxel (NV) and global voxel (GV) modules, which not only extract single voxel details but also consolidate global voxel insights, as depicted in Figure 3.

First, calculate the center-of-mass coordinates of all points within each voxel to extract the features of the point. Then, consider any voxel as a sub-voxel, calculate the feature weights within it, and establish a similarity analysis with its neighboring voxels to calculate the similarity weights. Finally, establish a mapping relationship between each local voxel and the entire point cloud space, as depicted in Equations (2) and (3). This allows the voxel feature extraction network to extract the information of a single voxel while simultaneously summarizing the voxel information of the entire domain. This approach enables a comprehensive and detailed feature expression of the object point cloud data, thereby improving the accuracy and completeness of feature extraction.

(2) $$F_{V}\left(p_{i}\right)=v_{j}$$

(3) $$F_{P}\left(v_{j}\right)=\{p_{i}|\forall p_{i}\in v_{j}\}$$

$p_{i}$ and $v_{j}$, respectively, represent the sets of point clouds and voxels, while $F_{P}\left(v_{j}\right)$ and $F_{P}\left(v_{j}\right)$ represent the corresponding feature mappings.

Based on the original channel attention mechanism, learnable parameters are introduced to regulate the concentration of the Gaussian distribution, thus enhancing the flexibility of the model. Short-range contextual information units are acquired when generating the Gaussian deviation matrix, which strengthens the network’s ability to extract key point information. This enables faster processing of voxel sequences, while dynamically adjusting based on the statistical information of the input sequences. The improved Gaussian deviation formula is shown in Equation (4):

(4) $$Attention=soft\max\left(\frac{-{\left(x-\left(\mu +w_{\mu }\right)\right)}^{2}}{2{\left(w_{\sigma }\sigma \right)}^{2}}\right)$$

$w_{\mu }$ and $w_{\sigma }$ are the parameters that need to be learned, allowing the model to adjust the mean and standard deviation.

The voxels traverse throughout the entire point cloud space, and obtain the value of α through model training to obtain the high-dimensional voxel feature mapping, as represented by Equation (5):

(5) $$F_{P}=\alpha v_{i}+(1-\alpha )v_{j}$$

$F_{P}$ represents high-dimensional voxel features, and α represents weight coefficients.

### 3.2. LiDAR and Image Feature Fusion Module

**Figure 4:**
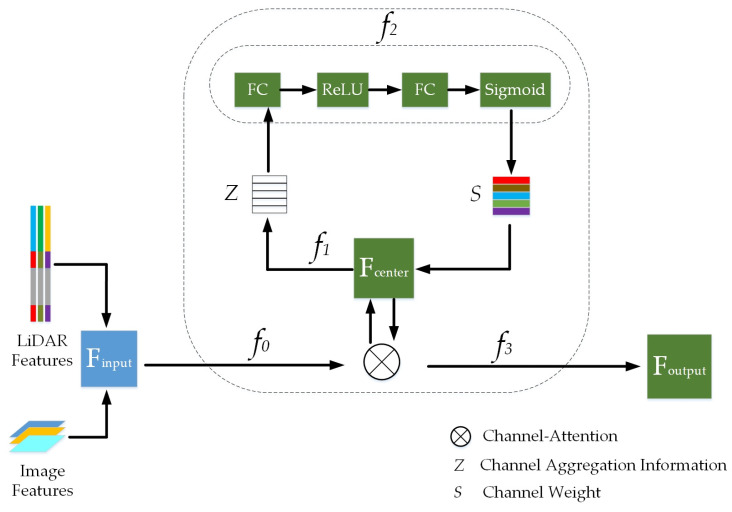
A series of convolution operations. $F_{input}$ represents the joint feature map of LiDAR and camera, which is transformed to the middle feature map $F_{center}$ through $f_{0}$. By sequentially performing transformations $f_{1}$, $f_{2}$, and $f_{3}$ on $F_{center}$, different-weighted feature maps $F_{output}$ can be outputted.

Using this process, this paper improves the channel attention mechanism model. In the convolutional layer, a dual convolution fusion operator is added. It first determines the fine-grained regions in the object category labels, then divides them into local regions to form a multi-region fine-grained fusion layer. Next, dual-layer convolution operations are performed separately on each fine-grained region to obtain a multi-region fine-grained feature fusion map. Finally, the obtained fusion feature map is nonlinearly mapped using the ReLU function, and low-dimensional and sized feature maps are outputted through the pooling layer. The attention fusion module of this paper integrates point cloud and image features at different stages of the entire network, and the total feature output is a concatenation with information weights for each part. The specific method is shown in Figure 4.

**Figure 5:**
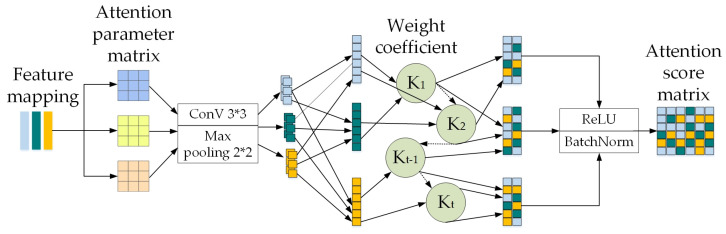
Voxel feature augmentation module. When the original voxelized network extracts features point by point, NV and GV units are added, and optimized voxel features are obtained after aggregating both features by the channel attention mechanism and finally spliced with the original voxelized features.

The model first conducts a detailed analysis of the channel dimension of each feature map; then, it adaptively learns a corresponding weight coefficient, and finally weights the features on the channel, allowing the network to decide which channel’s features to focus on more during the learning process, as shown in Figure 5.

#### 3.2.1. Calculating the Correlation Values

This section calculates the correlation values between point cloud features and image features. The specific calculation formula is shown in Equations (6) and (7).

(6) $$R_{I}=\sigma \left(F_{P}\cdot \left(W_{I}F_{I}+b_{I}\right)\right)$$

(7) $$R_{P}=\sigma \left(F_{I}\cdot \left(W_{P}F_{P}+b_{P}\right)\right)$$

$W_{P}$ and $W_{I}$ are the trainable parameter matrices trained for the point cloud and image networks, respectively. $F_{I}$ represents multi-scale image features. $b_{I}$ and $b_{P}$ correspond to the bias vectors of the image and point cloud features. σ is the ReLU function, which performs numerical transformation on the dot product of the two feature mappings to obtain the correlation function value.

#### 3.2.2. Calculating the Attention Scores 

This section calculates the attention scores of voxels encoding sequences for each image feature index. The specific calculation formula is shown in Equations (8) and (9).

(8) $$Att_{I}=soft\max(R_{I})$$

(9) $$Att_{P}=soft\max(R_{P})$$

$Att_{I}$ and $Att_{P}$ correspond, respectively, to the shared attention scores between dual semantic features. The softmax function is used to normalize the correlation function values between point cloud features and image features.

#### 3.2.3. Calculating the Final Output Total Feature 

This section calculates the total feature mapping of the point cloud and image. The specific calculation formula is shown in Equations (10) and (11).

(10) $$M_{ij}=\frac{\exp\left(D_{i}\times D_{j}\right)}{\sum_{i=1}^{N}\exp\left(D_{i}\times D_{j}\right)}$$

(11) $$F_{output}=\lambda \sum_{i=1}^{N}\left(M_{ji}D_{i}\right)+D_{j}$$

i and j represent different channels, $D_{i}$ and $D_{j}$ represent the original matrices, $F_{output}$ represents the total feature mapping of the point cloud and image. $M_{ij}$ represents the weighted sum of features for all channels, N represents the number of sequence groups, and λ is the learnable weight allocation parameter.

## 4. Experiments 

In this section, we first describe the implementation details. Subsequently, our proposed DL-VFFA is evaluated by presenting the experimental results on the KITTI [39] dataset, and ablation experiments are performed to verify the effectiveness of each module.

### 4.1. Implementation Details

#### 4.1.1. Dataset Setup and Environment Configuration

Considering the complexity of the model and the large number of samples used in our study, we have opted for the 0.7:0.3 partitioning ratio in the KITTI dataset. This ensures an ample amount of data for the training set to learn the model’s features while also providing sufficient data for validating the model’s generalization capability. After the split, the training set consisted of 5239 samples, while the validation set consisted of 2242 samples. Evaluation was conducted based on different levels of difficulty, including easy, moderate, and hard, determined by variations in size, occlusion, and truncation. We selected three classes of recognition from the KITTI labels: cars, pedestrians, and cyclists. The experiments were conducted on Ubuntu 18.04 LTS using an NVIDIA GeForce GTX 3060 GPU (NVIDIA, Santa Clara, CA, USA). We utilized the open-source 3D detection toolkit mmdetection3D. The practical virtual environment was established using Python 3.8.8 and PyTorch 1.8.0, with CUDA version 11.1. We employed the visualization software open3D 0.12.0 to visualize point cloud images.

#### 4.1.2. Detector Detail Settings

To ensure that the lighting conditions in the dataset do not affect the results, we performed post-processing on the images in both the training and testing sets before conducting the experiments, thereby reducing the impact of lighting on the images. The image feature extraction takes an image with a resolution of 1280 × 384 as input and outputs feature maps of four modules, which have dimensions of 256, 512, 1024, and 2048, respectively. The FPN is used as an image neck network to output 256-dimensional multiscale features, and the training dataset is expanded by adding random noise and flipping. The network is trained using stochastic gradient descent with a learning rate of 0.0005 and a momentum of 0.9. For the LiDAR point cloud, the ranges along the X, Y, and Z axes are [0, 70.4], [−40, 40], and [−3, 1] meters, respectively, and the voxel sizes are set to [0.05, 0.05, and 0.1] meters. The dynamic VFE extracts 64-dimensional voxelized features from the original features. The backbone Second network is trained for 20 epochs, optimized with the ADAM optimizer and a single-cycle learning rate strategy, with weight decay set to 0.01, division factor set to 10, momentum ranging from 0.95 to 0.85, and a maximum learning rate of 0.003.

### 4.2. Experiment Results

**Figure 6:**
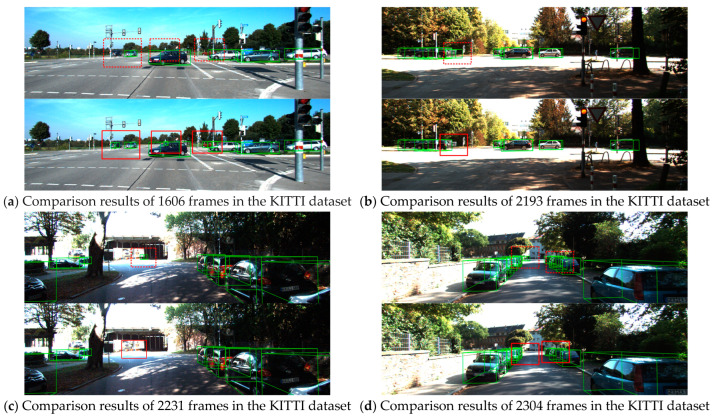
Some examples from the KITTI test dataset (**a**–**d**). The green bounding box represents the detection results of objects, the red dashed rectangular box represents false and missed detections, and the red solid rectangular box represents the correct detection results. Compared to MVX-Net, the optimized voxel fusion module in DL-VFFA can further enhance the key point feature information in the local details of challenging objects during detection, thus achieving better detection performance.

**Figure 7:**
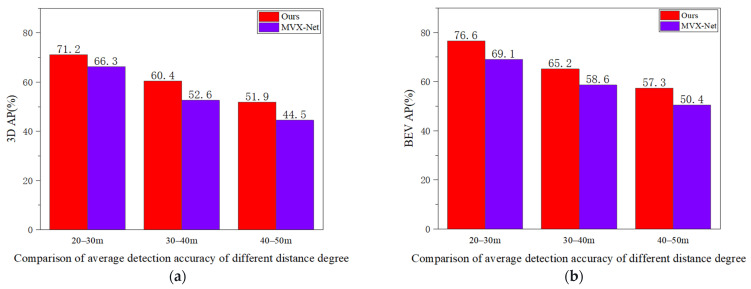
Under both the 3D perspective (**a**) and the BEV perspective (**b**), the comparative results of our method with MVX-Net under different distance degrees.

**Figure 8:**
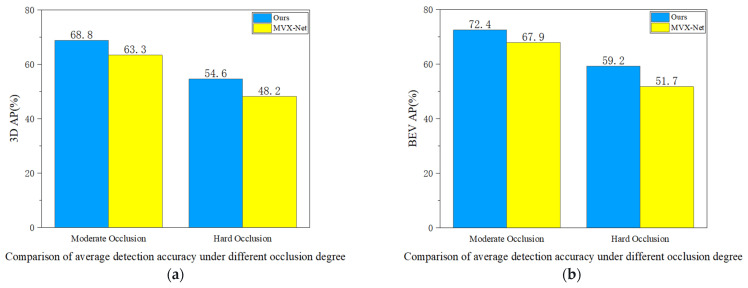
Under both the 3D perspective (**a**) and the BEV perspective (**b**), the comparative results of our method with MVX-Net under different occlusion degrees.

We compared DL-VFFA with other mainstream multimodal fusion networks based on the KITTI dataset, and Table 1 shows the detection results of the KITTI dataset. Through comparative analysis, DL-VFFA achieved better results in detecting objects in distant scenes and in scenarios with occlusions. The specific details are as follows:

(1) In Figure 6a, we correctly detected the red vehicle missed by MVX-Net near the distant traffic signal post. In the examples of Figure 6b,c, MVX-Net mistakenly identified a distant billboard as a vehicle, while our proposed network accurately filtered it out. In the example of Figure 6d, the network not only performed well in detecting nearby vehicles but also accurately detected the vehicles mistakenly identified by MVX-Net at distant scenarios.

(2) As shown in Figure 7, we selected objects from the KITTI dataset at different distance levels to compare the performance of the two algorithms in detecting small objects. In terms of 3D Average Precision (3D-AP) and Bird’s Eye View Average Precision (BEV-AP), compared to MVX-Net, our method achieves an average precision increase of 4.9% and 7.5%, respectively, at the distance level of 20–30 m; at the distance level of 30–40 m, the average precision increases by 7.8% and 6.6%, respectively, compared to MVX-Net; at the distance level of 40–50 m, the average precision increases by 7.4% and 6.9%, respectively, compared to MVX-Net.

(3) As shown in Figure 8, we selected objects from the KITTI dataset with different levels of occlusion to compare the voxel feature enhancement performance of the two algorithms under occluded conditions. In terms of 3D-AP and BEV-AP, compared to MVX-Net, our method achieves average precision increases of 5.5% and 4.5%, respectively, at the moderate occlusion level; at the severe occlusion level, the average precision increases by 6.4% and 7.5%, respectively, compared to MVX-Net.

### 4.3. Ablation Studies

**Figure 9:**
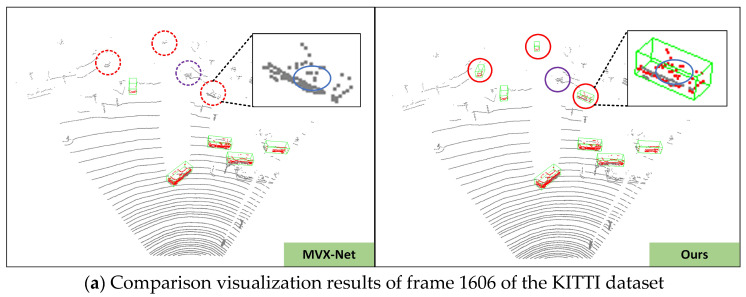

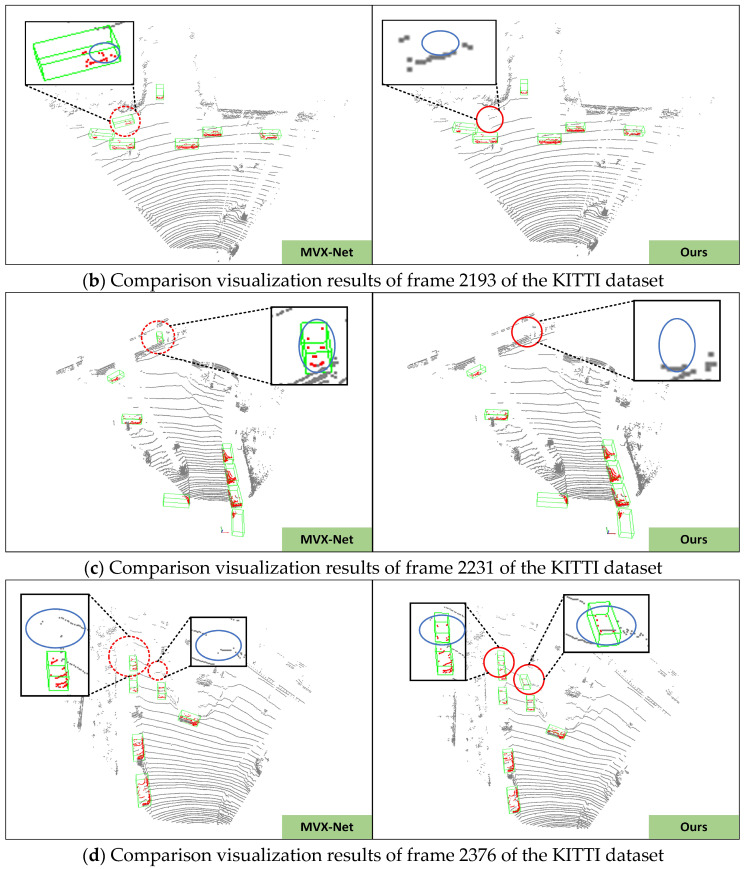
Qualitative analysis of DL-VFFA results on the KITTI dataset. The (**left side**) shows the detection results of MVX-Net, while the (**right side**) displays our method. Green bounding boxes represent detected objects; points in the point cloud are marked in red; red dashed lines and circles indicate differences between MVX-Net and our method. Blue solid ellipse boxes illustrate changes in the number and location of point clouds during voxelization for both detection networks.

To validate the effectiveness of DL-VFFA and explore the differences brought by its implementation details, we designed ablation experiments for different improved modules of DL-VFFA. Additionally, we selected four prominent voxel fusion enhancement networks as baseline models to evaluate DL-VFFA’s enhancement effect on other baseline networks. These four baseline networks are MVX-Net, SFD [40], V2PNet, and G-Fusion [41]. Given that our proposed method primarily focuses on refining MVX-Net, we compared the voxel fusion effects of DL-VFFA with those of MVX-Net and visualized four scenarios to ensure a more intuitive demonstration of the improved voxel fusion effects, as depicted in Figure 9. In this section, we used mean average precision (mAP) and frames per second (FPS), which are commonly used in KITTI datasets, as evaluation indices for ablation experiments. Intersection over Union (IOU) is a common metric used to evaluate the performance of object detection tasks. It measures the overlap between detection results and ground truth annotations. Specifically, IOU is calculated by dividing the area of overlap between the bounding boxes of the detection results and the ground truth annotations by the area of their union. The value of IOU ranges from 0 to 1, with a value closer to 1 indicating a higher degree of overlap between the detection result and the ground truth annotation, indicating better detection performance. We evaluated them on the KITTI test set, and the results of the ablation experiments are shown in Table 2, Table 3 and Table 4.

#### 4.3.1. Quantitative Analysis

We used Second as the baseline network to evaluate the 3D detection performance of DL-VFFA on the KITTI dataset. As shown in Table 2, adding the N-GV module resulted in a 1.37% improvement in mAP compared to the baseline network Second, with only a 0.05 s increase in inference time. With the addition of the GDM module, our mAP increased by 1.74% compared to the Second baseline network, with only a 0.03 s increase in inference time. Similarly, with the inclusion of the LWP module, our mAP improved by 1.46% compared to the baseline network Second, while the inference time decreased by 0.04 s. As shown in Table 3, when IOU = 0.5, DL-VFFA achieved a mAP of 79.48% in voxel fusion, and when IOU = 0.7, DL-VFFA achieved a mAP of 72.25% in voxel fusion. These represent improvements of 3.21% and 1.54%, respectively, over MVX-Net. The model ran at a speed of 11.0 frames per second, which is 0.8 FPS higher than MVX-Net. DL-VFFA maintains good real-time performance while achieving higher detection accuracy in voxel feature fusion. As shown in Table 4, in 3D view and BEV view, compared to the baseline network SFD, DL-VFFA’s mAP increased by 3.61% and 2.89%, respectively; compared to the baseline network V2PNet, DL-VFFA’s mAP increased by 2.05% and 1.84%, respectively; compared to the baseline network G-Fusion, DL-VFFA’s mAP increased by 2.77% and 3.40%, respectively. Experimental data show that the N-GV module enhances voxel features of point clouds, effectively utilizing local detail information of each voxel and significantly reducing the probability of false negatives and false positives for challenging objects. The GDM module dynamically adjusts position encoding based on input voxel sequences, enhancing the correlation between voxel sequences. The LWP module eliminates feature interference from redundant voxels without adding too many model parameters, thus accelerating the processing speed of the voxel sequences.

#### 4.3.2. Qualitative Analysis

In this section, we visualized some scenes from the KITTI dataset to intuitively observe the comparative effects of voxel feature fusion modules between MVX-Net and DL-VFFA. The specific analysis is as follows: In Figure 9a, MVX-Net missed some cars in distant scenes (indicated by red circles). DL-VFFA optimized this part, but the vehicles within the purple circles remained undetected, which may be due to issues in calculating the weights of image and point cloud features. This will be optimized in future work. In Figure 9b,c, MVX-Net incorrectly identified billboards as vehicles and generated incorrect detection bounding boxes, while DL-VFFA accurately filtered them out. In Figure 9d, even with severe obstruction between distant vehicles, DL-VFFA achieved accurate recognition. Due to occlusion between objects and their small size in the field of view, the original point clouds of the objects are very sparse or even overlapped, causing the detection network to lose local detail information during voxelization, making it difficult to distinguish between objects. Under the premise of keeping the original point cloud unchanged, after voxel fusion processing, the number and position of point clouds within the missed object bounding boxes changed significantly, indicated by blue elliptical bounding boxes in the figure. This demonstrates that the voxel features fusion method proposed in this paper can effectively allocate point clouds of the same objects to corresponding voxel units and calculate the correlation between voxel feature sequences using an improved Gaussian deviation matrix, thereby effectively reducing false positives and false negatives in vehicle detection.

## 5. Conclusions

We propose a dual-layer voxel feature fusion augmentation network for object detection. On the one hand, it utilizes an improved voxel-net to extract point cloud features and further enhances the detailed features of voxel sequences. On the other hand, an improved channel attention mechanism is employed to fuse semantic information from images and point clouds. This enables these two feature mappings to interact adaptively through weight coefficient matrices, thereby enhancing the detection network’s accuracy in scenarios with occlusion or distant objects. Experimental results demonstrate that in challenging scenarios on the KITTI dataset, the three improved modules in DL-VFFA achieve increases of 1.37%, 1.74%, and 1.46% in object detection mean average precision (mAP) compared to the baseline network Second. Under the 3D perspective, DL-VFFA achieves an object detection average precision of 76.40%, representing an improvement of 4.86% over MVX-Net. Under the BEV perspective, DL-VFFA achieves an object detection average precision of 78.51%, representing an improvement of 4.63% over MVX-Net. The voxel fusion method in DL-VFFA outperforms the voxel fusion module in MVX-Net by 1.54%(IOU = 0.7) in object detection mean average precision (mAP) while also reducing the inference time of the voxel sequence fusion model. This demonstrates that DL-VFFA effectively addresses the issue of misrecognition caused by occlusion or distant objects. Our research provides a more accurate and reliable object detection system, which contributes to enhancing road safety and traffic efficiency. This is crucial for the safety of modern and future vehicles, as it can reduce traffic accident rates and minimize casualties and vehicle losses. Additionally, it drives the development of autonomous driving technology and intelligent traffic systems, bringing more innovation and progress to the future of transportation. In future work, to enhance the all-weather perception capability of the object detection system, further improvement in target detection accuracy under low-light conditions is necessary.

## Figures and Tables

**Table 1:** Comparison results on the KITTI test dataset. The highlighted areas represent the results based on our method.

Method	3D AP (%)			BEV AP (%)		
	Easy	Mod.	Hard	Easy	Mod.	Hard
Second	84.91	72.65	43.96	85.38	73.87	54.53
Voxel-Net	81.62	65.23	50.77	82.26	66.73	52.29
AVOD	74.96	63.55	53.70	76.42	64.81	54.92
PointPainting	89.53	73.89	55.74	91.36	75.23	58.37
MVX-Net	85.52	72.26	56.83	88.63	73.61	59.41
SFD	87.56	77.61	60.95	88.12	80.06	62.24
V2PNet	84.33	76.35	60.28	85.69	77.83	62.31
G-Fusion	86.37	76.43	60.54	87.49	77.52	61.09
Ours	87.79	79.92	61.48	88.95	82.30	64.28

**Table 2:** Effectiveness of different modules of DL-VFFA.

Second	N-GV	GDM	LWP	mAP (%)	Inference Time (s)
1	0	0	0	66.46	0.88
1	1	0	0	67.83 (+1.37)	0.93 (+0.05)
1	0	1	0	68.20 (+1.74)	0.91 (+0.03)
1	0	0	1	67.92 (+1.46)	0.84 (−0.04)
1	1	1	0	68.95 (+2.49)	0.95 (+0.07)
1	1	0	1	69.23 (+2.77)	0.96 (+0.08)
1	0	1	1	68.65 (+2.19)	0.93 (+0.05)
1	1	1	1	70.39 (+3.93)	1.07 (+0.19)

**Table 3:** Comparative test results of voxel feature fusion module between MVX-Net and DL-VFFA.

Method	mAP (%) IOU = 0.5	mAP (%) IOU = 0.7	FPS
MVX-Net	76.27	70.71	10.2
Ours	79.48	72.25	11.0

**Table 4:** Cooperating with different baseline detectors.

Method	With DL-VFFA	3D AP (%)			BEV AP (%)		
		Easy	Mod.	Hard	Easy	Mod.	Hard
SFD	No	87.56	77.61	60.95	88.12	80.06	62.24
	Yes	89.24	81.67	66.05	90.35	82.30	66.43
	Improvement	+1.68	+4.06	+5.10	+2.23	+2.24	+4.19
V2PNet	No	84.33	76.35	60.28	85.69	77.83	62.31
	Yes	86.82	77.94	62.36	87.56	79.20	64.58
	Improvement	+2.49	+1.59	+2.08	+1.87	+1.37	+2.27
G-Fusion	No	86.37	76.43	60.54	87.49	77.52	61.09
	Yes	88.21	79.06	64.37	89.78	80.63	65.87
	Improvement	+1.84	+2.63	+3.83	+2.29	+3.11	+4.78

## Data Availability

Data are contained within the article.
